# Large language models for generating medical examinations: systematic review

**DOI:** 10.1186/s12909-024-05239-y

**Published:** 2024-03-29

**Authors:** Yaara Artsi, Vera Sorin, Eli Konen, Benjamin S. Glicksberg, Girish Nadkarni, Eyal Klang

**Affiliations:** 1 Azrieli Faculty of Medicine, Bar-Ilan University, Ha’Hadas St. 1, Rishon Le Zion, Zefat, 7550598 Israel; 2 Department of Diagnostic Imaging, Chaim Sheba Medical Center, Ramat Gan, Israel; 3 Tel-Aviv University School of Medicine, Tel Aviv, Israel; 4 DeepVision Lab, Chaim Sheba Medical Center, Ramat Gan, Israel; 5 Division of Data-Driven and Digital Medicine (D3M), Icahn School of Medicine at Mount Sinai, New York, NY USA; 6 The Charles Bronfman Institute of Personalized Medicine, Icahn School of Medicine at Mount Sinai, New York, NY USA

**Keywords:** Large language models, Generative pre-trained transformer, Multiple choice questions, Medical education, Artificial intelligence, Medical examination

## Abstract

**Background:**

Writing multiple choice questions (MCQs) for the purpose of medical exams is challenging. It requires extensive medical knowledge, time and effort from medical educators. This systematic review focuses on the application of large language models (LLMs) in generating medical MCQs.

**Methods:**

The authors searched for studies published up to November 2023. Search terms focused on LLMs generated MCQs for medical examinations. Non-English, out of year range and studies not focusing on AI generated multiple-choice questions were excluded. MEDLINE was used as a search database. Risk of bias was evaluated using a tailored QUADAS-2 tool.

**Results:**

Overall, eight studies published between April 2023 and October 2023 were included. Six studies used Chat-GPT 3.5, while two employed GPT 4. Five studies showed that LLMs can produce competent questions valid for medical exams. Three studies used LLMs to write medical questions but did not evaluate the validity of the questions. One study conducted a comparative analysis of different models. One other study compared LLM-generated questions with those written by humans. All studies presented faulty questions that were deemed inappropriate for medical exams. Some questions required additional modifications in order to qualify.

**Conclusions:**

LLMs can be used to write MCQs for medical examinations. However, their limitations cannot be ignored. Further study in this field is essential and more conclusive evidence is needed. Until then, LLMs may serve as a supplementary tool for writing medical examinations. 2 studies were at high risk of bias. The study followed the Preferred Reporting Items for Systematic Reviews and Meta-Analyses (PRISMA) guidelines.

**Supplementary Information:**

The online version contains supplementary material available at 10.1186/s12909-024-05239-y.

## Background

There is a global shortage of clinical practitioners and increasing demand for medical professionals. This need presents significant challenges in the healthcare system [1–3]. In response, the number of medical schools and students has been rising worldwide [4, 5], leading to an increase in the demand for written tests.

Multiple choice questions (MCQs) are considered popular for testing applied knowledge in the basic and clinical sciences [6]. When constructing good quality MCQ, the agreed upon model comprises a stem, the initial part of the question, which is clearly written, containing all the information necessary to answer the question. The lead-in question contains only one answer that is clearly the best choice, followed by a number of optional answers called “distractors”. The distractors should be plausible to those without detailed knowledge of the subject, reducing the chance of guessing the correct answer [7]. MCQs should cover a broad range of the curriculum and be representative of the material that students are expected to learn.

The item difficulty, i.e. difficulty level of the MCQs, should also be appropriate for the level of the learner. They should be challenging enough to discriminate between those who understand the material and those who do not, but not so difficult as to be discouraging. Good MCQs should be able to discriminate between higher and lower performing students so that students who perform well on the overall exam should be more likely to answer the question correctly than those who perform poorly [8, 9, 13].

Creating multiple choice questions (MCQs) requires medical knowledge, conceptual integration, and avoiding potential pitfalls, for example, repeating the same MCQs in examinations from year to year, rendering the question less useful, or inherent imperfections called item-writing flaws (IWFs). A study by Rush et al. details some of the more common writing flaws, including mutually exclusive distractors, where students can recognize that one of the two mutually-exclusive responses is correct, thus eliminating other options. Another common IWF is “longest answer is correct”, a common issue made by examination writers in an effort to ensure the correct response is indisputable, or use of absolute terms (always, never, all). Students recognize that absolute terms usually render a statement false [10]. While IWFs may appear trivial, they can affect the way students understand and answer questions [10–13]. Producing MCQs is also time consuming, and any application capable of automating this process could be highly valuable for medical educators [14, 15].

Amidst these challenges, advancements in natural language processing (NLP) are constantly discussed and evaluated [16], in particular, the introduction of OpenAI’s state-of-the-art large language models (LLMs) such as GPT-3.5 and GPT-4 [17, 18]. These models offer potential solutions to healthcare education, due to their human-like text understanding and generation, which includes clinical knowledge [19]. This could be pivotal in automating the creation of medically precise MCQs.

According to Bond et al. another possible application of AI in medical education is grading patients notes. This can provide additional formative feed-back for students in the face of limited faculty availability [20].

AI based technologies are continuously evolving, becoming more popular in medical education. One such technology is Virtual Patients (VP), which are interactive computer simulations of real-life clinical scenarios. They are used for medical training, education and assessment. By using AI to provide realistic patient interactions, students can practice clinical decision-making and also receive feedback in a safe and controlled environment [21].

Medical knowledge is continually and rapidly evolving; therefore, up-to-date medical questions generation may be hard to keep up with for medical educators [22]. Automatically generated MCQs could be quicker to implement when medical knowledge changes current practices, or when new discoveries and forefronts are reached. Automated MCQs could also assist medical students in practicing learning material with a vast data resource, which can supply a limitless amount of MCQs in a short amount of time [23]. Moreover, automated MCQs generation can tailor a personalized learning experience which can provide students with a formative assessment. Formative assessments allow for feedback which improves learning, while summative assessments measure learning. Formative tests were shown to improve classroom practice, and encourage students in both reflective and active review of learning material. In general terms, formative assessment assists students in developing their learning skills [20, 24, 25].

However, automating MCQs creation introduces potential risks, as the accuracy and quality of AI generated content is still in question [26, 27]. We aimed to review the literature on LLMs’ ability to generate medical questions. We evaluated their clinical accuracy and suitability for medical examinations in context of their limitations.

## Methods

### Literature search

On November 2nd 2023 we conducted a search identifying studies describing LLMs’ applications in generating medical questions. Since the Chat-GPT LLM launched by OpenAI on November 30, 2022, we limited our search period to 2023. We searched PubMed/MEDLINE for papers with the following keywords, using Boolean operators AND/ OR: large language models; GPT; Chat-GPT; medical questions; medical education; USMLE; MCCQE1; board exam; medical exam. We also checked the references list of selected publications for more relevant papers. Sections as ‘Similar Articles’ below articles (e.g., PubMed) were also inspected for possible additional articles.

#### Ethical approval

was not required, this is a systematic review of previously published research, and does not include any individual participant information. Our study followed the Preferred Reporting Items for Systematic Reviews and Meta-Analyses (PRISMA) guidelines. The study is registered with PROSPERO (CRD42023481851).

### Inclusion and exclusion process

**Fig. 1:**
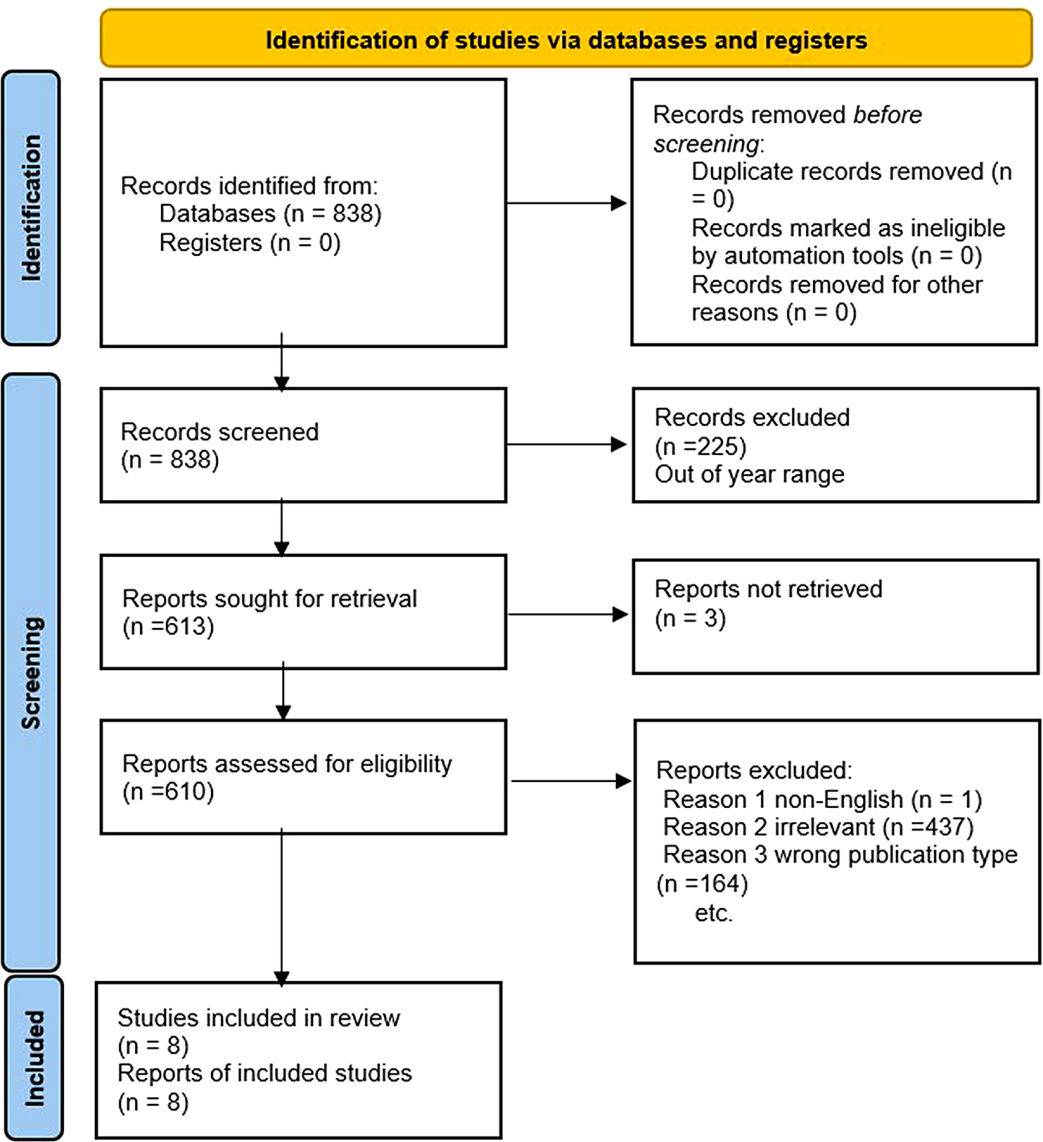
Flow diagram of the search and inclusion process in the study. Flow Diagram of the Inclusion Process. Flow diagram of the search and inclusion process based on the Preferred Reporting Items for Systematic Reviews and Meta-Analyses (PRISMA) guidelines, November 2023

Publications resulting from the search were initially assessed by one author (YA) for relevant titles and abstracts. Next, full-text papers underwent an independent evaluation by two authors (EK and VS) (Fig. 1).

**Fig. 2:**
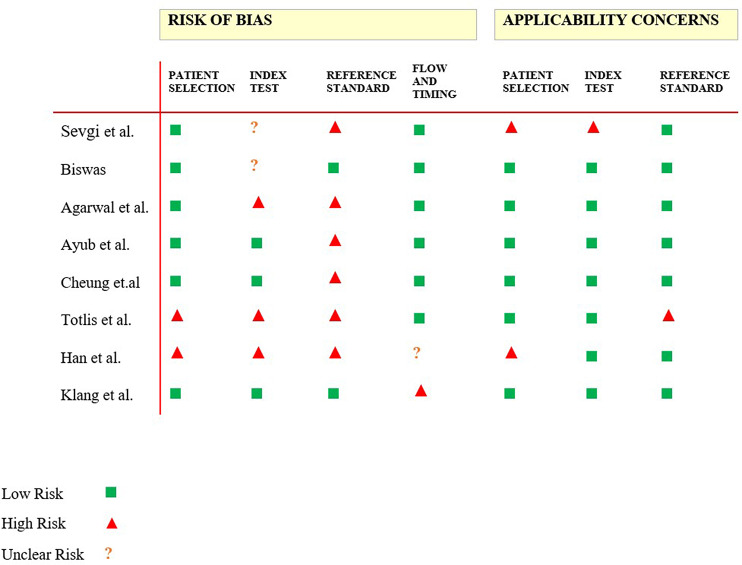
Risk of Bias and Applicability Judgments in QUADAS-2. QUADAS-2 table for potential bias and applicability. Risk of bias and applicability were evaluated using the tailored QUADAS-2 tool, November 2023

We included full length studies describing LLMs generating medical questions published no earlier than 2023. Exclusion criteria included: (1) non-English language, (2) wrong publication type (e.g. review article, case reports and case series, editorial and opinion pieces, commentaries and letters to the editor, conference abstracts and presentations, technical reports and white papers, book chapters and monographs), (3) publication year out of range (4), Full-text not available, (5) duplicates, (6) no MCQ generation by AI. Any study in question was discussed among all authors until reaching a unanimous agreement. Risk of bias and applicability were evaluated using the tailored QUADAS-2 tool (Fig. 2).

Risk of bias and applicability were evaluated using the QUADAS-2 tool. (Fig. 2).

## Results

### Study selection and characteristics

**Fig. 3:**
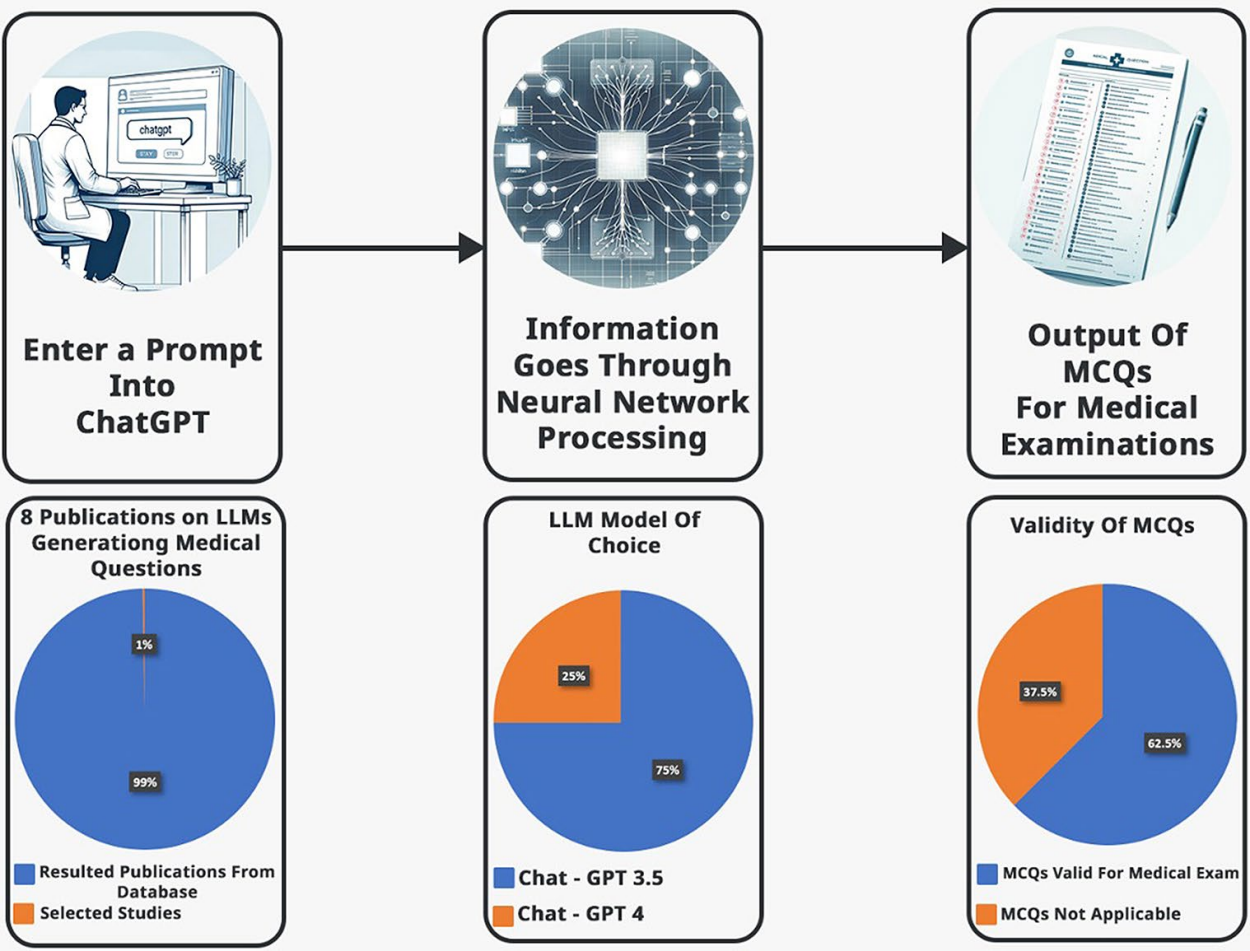
Illustration of multiple-choice questions (MCQs) generation and summary of preliminary results. A graphical illustration of MCQs generation and preliminary data. Upper row images were created using Chat-GPT 4 and DALI, illustrating the MCQs generation process via a large language model. The images created in the bottom row showcase preliminary data results, November 2023

The initial literature search resulted in 838 articles. Eight studies met our inclusion criteria (Fig. 1). Most studies were retrospective: 6/8 (75%). One study is cross-sectional and one study is prospective. Most studies used Chat-GPT (3.5 or 4) as an AI model of choice, other models evaluated included Microsoft’s Bing and Google’s Bard. The MCQs were produced with varying parameters (Table 1). Overall, 5/8 (62.5%) studies demonstrated valid MCQs. 6/8 (75%) of the studies utilized the latest version Chat-GPT 4 (Fig. 3.)

**Table 1:** General features of the articles in the study

Study	Author	Month	Journal	Study design	AI tool
1	Sevgi et al.	April	Neurosurgical Review	Retrospective	Chat-GPT 3.5
2	Biswas	May	Annals of Biomedical Engineering	Retrospective	Chat-GPT 3.5
3	Agarwal et al.	June	Cureus	Cross-sectional study	Chat-GPT, Bard, Bing
4	Ayub et al.	August	Cureus	Retrospective	Chat-GPT 3.5
5	Cheung et al.	August	PLOS ONE	Prospective	Chat-GPT 3.5 plus
6	Totlis et al.	August	Surgical and Radiologic Anatomy	Retrospective	Chat-GPT 4
7	Han et al.	October	Medical Teacher	Retrospective	Chat-GPT 3.5
8	Klang et al.	October	BMC Medical Education	Retrospective	Chat-GPT 4

Summary of the articles in the literature that applied AI for generating medical questions, November 2023

### Descriptive summary of results

Cheung et al. [28] were the first, and so far, the only study to compare LLM to humans in MCQs writing. Chat-GPT 3.5 plus generated the MCQs. The reference for the prompt were two standard undergraduate medical textbooks: Harrison’s Principles of Internal Medicine the 21th edition for medicine [29], and Bailey and Love’s Short Practice of Surgery 27th Edition for surgery [30]. Only four choices were given per question. Also, only text and knowledge-based questions were generated. No modification to the MCQs was allowed after generation. Chat-GPT 3.5 performed relatively well in the task. The overall time required for the AI to generate 50 MCQs was 21 min. This is about 10% of the total time human writing required (211 min). However, the questions written by humans were far better. Both in terms of quality and validity, outperforming the AI in a total score of 30 (60%) eligible MCQs (Table 2).

**Table 2:** Key parameters investigated in each study

Author	No. of MCQs	Tested vs. Human	Medical Field	Questions Evaluated By	Performance Scores
Sevgi et al.	3	No	Neurosurgery	Evaluated by the author according to current literature	2 (66.6%) of the questions were accurate
Biswas	5	No	General	N/A	N/A
Agarwal et al.	320	No	Medical Physiology	2 Physiologists	**p value validity < 0.001 for**: Chat-GPT vs. Bing < 0.001 Bard vs. Bing < 0.001 **p value of difficulty < 0.006** Chat-GPT vs. Bing 0.010 Chat-GPT vs. Bard 0.003
Ayub et al.	40	No	Dermatology	2 board certified dermatologists	16 (40%) of questions valid for exams
Cheung et al.	50	Yes	Internal Medicine/Surgery	5 International medical experts and educators	**Overall performance**: AI score 20 (40%) vs. Human score 30 (60%) Mean difference -0.80 ± 4.82 **Total time required**: AI 20 min 25 s vs. Human 211 min 33 s
Totlis et al.	18	No	Anatomy	N/A	N/A
Han et al.	3	No	Biochemistry	N/A	N/A
Klang et al.	210	No	Internal Medicine Surgery Obstetrics & Gynecology Psychiatry Pediatrics	5 Specialist physicians in the tested fields	**Problematic questions by field**: Surgery 30% Gynecology 20% Pediatrics 10% Internal medicine 10% Psychiatry 0%

Summary of key parameters investigated in each study, November 2023

Klang et al. [31] performed blind assessment of the generated questions. They did not disclose to the evaluators whether the MCQs origin was AI. At first, they asked Chat-GPT 4 to create MCQs on the topic of internal medicine. They used as reference (few-shot learning) a former exam of the same subject. The MCQs had four possible answers, with the correct answer marked with an asterisk. At first, the generated MCQs were short with no clinical background. This required a second prompting of the AI model, specifically requesting the AI to create MCQs with clinical history. The study showed promising results, with the majority of MCQs deemed valid as exam questions (Table 2).

In a cross-sectional study, Agarwal et al. [32] compared different LLMs. They compared Chat-GPT 3.5/Bard/Bing in MCQs generating capability. They used as reference the 11-module curriculum for physiology, created by The Indian National Medical Commission (NMC). The authors requested in the prompt to Generate five difficult reasoning-based MCQs, fitting levels of Bachelor of Medicine, and Bachelor of Surgery (MBBS). Chat-GPT’s generated MCQs were significantly more valid than the other AI tools examined in the study. However, the difficulty level was lower compared to Bard and Bing (Table 2).

Ayub et al. [33] focused on medical board examination for Dermatology. They utilized Chat-PDF to upload entire PDF files into a Chat-GPT 3.5 portal. The reference used was “Continuing medical education” (CME) articles, taken from the *Journal of the American Academy of Dermatology* (JAAD). This reference is considered high-yield review material for the American Board of Dermatology Applied Exam (ABD-AE). This study’s prompt was not detailed in the paper. The three parameters to evaluate the MCQs were accuracy, complexity, and clarity. Only 16 (40%) of the generated questions were applicable (Table 2). The rest were unclear 9 (22%), inaccurate 5 (13%) or had low complexity 10 (25%) (Table 3). Sevgi et al. [34] asked Chat-GPT 3.5 to prepare three questions with answers and explanations at a level appropriate for a neurosurgery board exam. There was no independent evaluation of the MCQs.

Han et al. [35] instructed Chat-GPT 3.5 to write three MCQs, each containing clinical background and lab values. Each time they requested Chat-GPT to rephrase the question. First, for a different correct answer and then for an increased level of difficulty. There was no independent evaluation of the MCQs.

Totlis et al. [36] asked Chat-GPT 4 to generate MCQs on the topic of anatomy. In the prompt they requested increasing difficulty and matching correct pairs. There was no independent evaluation of the MCQs. Biswas [37] requested in the prompt to prepare MCQs for USMLE step 1 exam. There was no independent evaluation of the MCQs.

All studies presented some faulty questions that were deemed inappropriate for medical exams. Some questions required additional modifications in order to qualify (Table 3). We included in additional files examples from each study, demonstrating valid MCQs as well as faulty MCQs for various reasons (Supplementary Table 1.)

**Table 3:** Present faulty questions generated by the AI

Author	Medically Irrelevant Questions	Invalid for Medical Exam	Inaccurate/Wrong Question	Inaccurate/Wrong Answer or Alternative answers	Low Difficulty Level
Sevgi et al.	N/A	N/A	N/A	1 (33.3%)	N/A
Biswas	N/A	N/A	N/A	N/A	N/A
Agarwal et al.	N/A	Highly valid	N/A	V/A	Somewhat difficult
Ayub et al.	9 (23%)	24 (60%)	5 (13%)	5 (13%)	10 (25%)
Cheung et al.	32 (64%)	28 (56%)	32 (64%)	29 (58%)	N/A
Totlis et al.	N/A	8 (44.4%)	N/A	N/A	8 (44.4%)
Han et al.	N/A	N/A	N/A	N/A	3 (100%)
Klang et al.	2 (0.95%)	1 (0.5%)	12 (5.7%)	14 (6.6%)	2 (0.95%)

Summary of faulty questions generated by the AI, November 2023

## Discussion

In this study we explored large language Models (LLMs)’ applicability in generating medical questions, specifically multiple choice questions (MCQs) for medical examinations. The studies we reviewed did not continue to test the generated MCQ in a real-world setting, i.e. with medical students. In order to truly evaluate the feasibility of LLMs application in the medical education field, this should be the next logical step.

MCQs are an essential component of medical exams, used in almost every aspect of medical education [12, 13], yet they are time consuming and expensive to create [38]. The possibility of AI generated questions can provide an important opportunity for the medical community and transform the way written tests are generated. Using LLMs to support these tasks can potentially save time, money, and reduce burnout, especially in a system already sustaining itself on limited resources [39].

### Benefits of AI-generated educational content

Burn-out, poor mental health, and growing personal distress are constantly studied in clinical practitioners [40]. However, academic physicians experience a unique set of additional challenges, such as increased administrative work, less time with patients, and increased clinical responsibilities. As a result, they have less time for traditional academic pursuits such as research and education [41–43]. In the famous words of Albert Einstein: “Bureaucracy is the death of any achievement”.AI can potentially relieve medical educators from tiresome bureaucracy and administrative work, allowing them to focus on the areas that they view as most personally meaningful and avoid career dissatisfaction [42, 44].

Moreover, AI-generated MCQs can assist medical students by creating personalized learning experience, while accessing current up-to-date information [45]. These are only a few examples of the benefits of AI in the generation of medical MCQs, and new areas for its utility are continuously discovered.

### Drawbacks of AI-generated educational content

Nowadays, AI continues to evolve, becoming more integrated in various medical fields [46]. AI performance is fast, efficient and with what seems like endless data resources [47]. In almost every study we reviewed, LLMs’ execution was more than satisfactory with the consensus that AI is capable of producing valid questions for medical exams. Presented here are examples for valid MCQs generated in the studies:

#### Example 01

“Which of the following is a negative symptom of schizophrenia?”

(A) Hallucinations.
(B) Delusions.
(C) Anhedonia.
(D) disorganized speech.

#### Example 02

“What is the anatomical term for the socket in the pelvic bone where the femur articulates?”

(A) Acetabulum.
(B) glenoid cavity.
(C) foramen magnum.
(D) fossa ovalis.

However, while these models show promise as an educational tool, their limitations must be acknowledged.

One notable limitation is a phenomenon known as “hallucination” [48]. This occurs in a wide variety of scenarios, resulting in outputs that lack logical consistency or completely unfactual information [49]. This phenomenon is unacceptable for MCQs. Issues in MCQs generation can arise from AI hallucinations and beyond, such as inappropriate MCQ complexity to the material, multiple correct answers and other inaccuracies. Presented here are examples for faulty MCQs generated by the AI:

#### Example 03

“Which of the following vessels is NOT a component of the Circle of Willis?”

(A) Anterior cerebral artery.
(B) Posterior communicating artery.
(C) Middle cerebral artery.
(D) Vertebral artery.
(E) Superior cerebellar artery.

In the above mentioned MCQ both D and E are correct.

#### Example 04

“Which of the following is a characteristic feature of melanoma?”

(A) Uniform color.
(B) Smooth borders.
(C) Symmetry.
(D) Irregular pigmentation.

The above-mentioned MCQ was deemed as low complexity for a standard exam, after a rigorous evaluation by a board-certified specialist in this field. The ability of AI to integrate contextual and sensory information is still not fully developed, as well as its understanding of non-verbal cues or body language. Furthermore, racial bias in medical education is a serious issue [50]. Inherent bias in data and inaccuracies of AI generated educational content is troubling, and could perpetuate a grave affliction of the medical education system [51, 52].

Another consideration is the logistics necessary to implement AI in healthcare and education. New technologies require training, commitment and investment in order to be maintained and managed in a sustainable way. Such a process can take time and energy [53]. In addition, careful consideration of prompt crafting must be a requisite for AI generated MCQs application in medical education. In each study, we examined the process of crafting the MCQs. We noticed a wide range of approaches to writing the prompts. In some studies, additional modifications took place in order to improve the validity of the questions. This emphasizes the importance and sensitivity of prompts, and the need for training educators and students in AI literacy and prompt engineering.

Prompt-engineering may be a task that requires specific training, so that the prompt is phrased correctly and the MCQs quality is not impaired. A good way for clinical practitioners and medical educators to enhance the quality of their prompts, is to first familiarize themselves with LLMs and understand the fundamentals of machine learning. General guidelines for optimizing prompts suggest trying to be as specific as possible, provide appropriate setting and context when phrasing the prompt, ask open ended questions, and request examples in order to clarify the meaning of a concept or idea [54]. A poor prompt for example is “Tell me about heart disease.” This prompt is not specific enough, and a good way to improve this prompt is to add details, for example “What are the most common risk factors for coronary artery disease?”

Particular concerns in regards to applications of AI in medical education are ethics and data privacy [55]. The current literature is limited on how to guide medical educators, ensuring that they are using AI ethically and responsibly in their teaching. Accordingly, awareness of the complexities of ethics and data privacy while using AI in medical education is called for. According to Masters (2023), these complexities include data gathering, anonymity and privacy, consent, data ownership, security, data and algorithm bias, transparency, responsibility, autonomy, and beneficence [56].

Equally important limitation of AI integration in education is accountability. The “black box” of AI models refers to the fact that much of the internal workings of the system are invisible to the user. Medical educators might use the AI to generate an exam, write the input and receive the output, but the system’s code or logic cannot be questioned or explained [57].

An additional aspect to consider is the longstanding concern of AI replacing human jobs, particularly within the medical workforce [58]. This thought process could cause resistance to AI utility and integration in clinical practice. This notion is unlikely in the near future and possibly ever. There is a quality to human interaction in care that cannot be replaced by machines. But, distrust in AI technology is yet another challenge to its implementation [59]. In light of this concern, it’s important to take into consideration medical educators and students’ perception of AI and LLMs on their application in medical education. Banerjee et al. examined postgraduate trainee doctors’ perception on the impact of AI on clinical education, with overall positive perception of AI technologies’ impact on clinical training [60].

In contrast, a recent study showed that even though AI is currently progressing towards clinical implementation, there was a lack of educational opportunities about AI in medicine among medical trainees [61]. When considering future research in this field, not only should the LLMs performance be studied, but also the understanding and acceptance of this technology among educational staff and students. There should be a continuous conversation about how humans and AI can work together, for instance in the sense of computer-aided diagnosis.

Perhaps one of the biggest concerns of AI application in medical education is impairing students’ critical thinking. According to Van de Ridder et al., self-reflection and criticism are crucial for a medical student’s learning process and professional growth. In a reality where a student can delegate to Chat-GPT tasks such as writing personal reflection or learning experiences, the students deny themselves of the opportunity to self-reflect and grow as physicians [62].

Lastly, all except for one study we examined [28], did not compare the AI generated MCQs with human written MCQs, and none of the studies tested the AI generated MCQs in a real-world setting, i.e., testing medical students. We believe this is the next required step in perfecting LLMs as a tool to assist in medical exam generation. A paper published after our search period by Laupichler et al. conducted this comparison in student performance in answering AI vs. human generated MCQs [63]. They found no statistically significant difference in item difficulty between AI generated MCQs and human generated questions, but discriminatory power was statistically significantly higher in humans than LLM questions.

Application of AI generated MCQs in medical education is still in its early stages. Although it shows much promise, it is imperative to take into consideration the significant shortcomings and challenges such application entails. AI should be used wisely and responsibly while integrating it into the medical education domain.

### Limitations

Our review has several limitations. Due to heterogeneity in study design and data, we were unable to perform a meta-analysis. Our search yielded a low number of results (eight). Only one author rated the initial results.

In addition, a notable limitation is the methodological quality of some of the analyzed studies. Most of the studies are retrospective in nature. Future longitudinal studies could help in understanding the long-term effectiveness and impact of LLMs in medical education. None of the questions were image or graph based, which is an integral part of medical exams. Three studies did not base their prompt on a valid medical reference, such as previous exams or approved syllabus. Three studies did not evaluate the questions after they were generated. Two studies were at high risk of bias.

We limited our search to PubMed/MEDLINE. Also, since Chat-GPT was launched by OpenAI on November 30, 2022, we restricted our search period to 2023. We did not expect to find relevant studies on the application of LLMs in medical education in earlier years. We acknowledge the fact that expanding the search could provide a more comprehensive overview of the development and use of LLMs in medical education.

Furthermore, we excluded non-English papers, thereby preventing a more global comprehensive perspective on cultural difference in LLMs application in education.

We recognize these choices narrow our review’s scope. This might exclude various relevant studies, possibly limiting diverse insights.

## Conclusion

AI-generated MCQs for medical exams are feasible. The process is fast and efficient, demonstrating great promise in the future of medical education and exam preparation. However, their use warrants cautious and critical evaluation. Awareness of AI limitations is imperative in order to avoid misuse and deterioration of medical education quality. We strongly suggest that further research should be conducted to determine the long-term effectiveness and impact of AI generated MCQs in comparison to traditional educational methods, as well as testing their acceptance and understanding among the medical education community. Until more advancements are achieved, AI should be viewed as a powerful tool best utilized by experienced professionals.

### Electronic supplementary material

Below is the link to the electronic supplementary material.

**Supplementary Material 1: Supplementary Table 1.** Examples from studies showcasing valid and faulty MCQs

**Supplementary Material 2:** PRISMA abstract 2020 checklist

**Supplementary Material 3:** Additional Files Legends

## Data Availability

All data generated or analyzed during this study are included in this published article and supplementary files.

## Abbreviations

LLM: Large language models
MCQ: Multiple choice question
GPT: Generative Pre-trained Transformer
IWF: Item writing flaw
AI: Artificial intelligence
VP: Virtual Patients
