# Supplementary material for: Large language models for generating medical examinations: systematic review
Source: BMC Med Educ. 2024 Mar 29;24:354. doi: 10.1186/s12909-024-05239-y (PMC10981304; doi:10.1186/s12909-024-05239-y)
Supplement: Supplementary file 3 — Supplementary Material 3: Additional Files Legends [file 12909_2024_5239_MOESM3_ESM.docx]

Additional Files

File name – Supplementary table 1

File format – Word docx

Title of data - Supplementary table 1. Examples from studies showcasing valid and faulty MCQs

Description of data - Examples from studies of multiple-choice questions generated by AI. Both valid and faulty, November 2023.

File name – PRISMA abstract checklist

File format – Word docx

Title of data – PRISMA abstract 2020 Checklist

Description of data – PRISMA abstract checklist for systematic review
